# More than Just a Brain Disorder: A Five-Point Manifesto for Psychological Care for People with Huntington’s Disease

**DOI:** 10.3390/jpm12010064

**Published:** 2022-01-07

**Authors:** Nicolò Zarotti, Maria Dale, Fiona J. R. Eccles, Jane Simpson

**Affiliations:** 1 Division of Health Research, Faculty of Health and Medicine, Lancaster University, Lancaster LA1 4YG, UK; nicolozarotti@gmail.com (N.Z.); f.eccles@lancaster.ac.uk (F.J.R.E.); 2 Mill Lodge Huntington’s Disease Inpatient and Community Service, Leicestershire Partnership NHS Trust, Leicester LE19 4SL, UK; maria.dale@leicspart.nhs.uk

**Keywords:** Huntington’s disease, psychological care, clinical psychology, psychotherapy, mental health

## Abstract

Huntington’s disease (HD) is a rare and complex condition where affected individuals, family members, caregivers, and clinicians face a number of both long-term and fluctuating challenges. The predominant biomedical framework adopted in HD to date has traditionally viewed it as a brain disorder first and foremost. As a consequence, one of the most challenging aspects of the condition—psychological difficulties and their care—is often not given the emphasis it deserves in everyday clinical practice. Here, we propose a manifesto outlining five points to address the quality, effectiveness, availability, and accessibility of psychological care in HD. These include (1) Listening to People with HD, (2) Reformulating Difficulties Psychologically, (3) Exploring New Interventions, (4) Increasing Psychological Provision, and (5) Learning from Other Conditions. As the search for a cure continues, we hope that this manifesto will create a new impetus towards refining the current approach to psychological difficulties in HD and ultimately improve the quality of life of the tens of thousands of families affected by HD worldwide.

## 1. Introduction

Since its earliest depiction, Huntington’s disease (HD) has been described as a neurodegenerative condition associated with a wide range of psychological issues. In his seminal 1872 article *On Chorea*, George Huntington explained that “as the disease progresses, the mind becomes more or less impaired, in many amounting to insanity, while in others mind and body gradually fail until death relieves them of their sufferings” [1] (p. 320). Today, we know that what Dr Huntington called “insanity” translates into a number of significant psychological difficulties experienced by people with HD (pwHD), which most frequently include low mood and mood extremes, irritability and aggressiveness, anxiety, agitation, perseveration, compulsions, apathy, avoidance, emotion dysregulation, and increased risk of suicide [2,3,4,5,6,7,8,9,10]. More rarely, pwHD may also experience obsessive–compulsive behaviours, delusions, and hallucinations [4,5,6]. HD is also associated with several cognitive impairments, which tend to involve mainly memory, attention, psychomotor speed, executive functioning and, later, language—ultimately leading to dementia [10,11].

After the discovery of the genetic mutation responsible for the disease in 1993 [12], genetic testing became available for individuals with a family history, allowing them to know if they carry the gene expansion even decades before the onset of symptoms. Based on this, individuals with a positive predictive genetic test but no motor symptoms are usually referred to as being ‘presymptomatic’ or ‘premanifest’ [11], while untested people with a family history of the disease are usually defined as ‘at-risk’ [13]. However, the results of a predictive test only allow confirmation of whether an individual carries the gene expansion and will therefore develop the disease; it does not pinpoint when onset will occur. This understandably turns the experience of predictive genetic testing into a further psychologically challenging aspect of the condition [14,15,16], with a number of reports showing that a positive test result can be associated with negative views about the future characterised by reduced social engagement, decreased long-term life planning, excessive hypervigilance or ‘symptom watching’, and increased suicidal ideation [17,18]. In turn, this may also explain the very low test uptake figures among at-risk individuals available in the literature (e.g., usually lower than 20% [19,20]).

Some of the hereditary and cognitive features of HD may also cause a number of psychosocial issues, which include genetic discrimination (i.e., being treated unfairly due to genetic differences), family tensions and overwhelming caregiver burden, guilt about passing HD on to children, living with a constant potential ‘window to the future’ when witnessing a parent’s decline, as well as impaired social affective skills (e.g., recognition of emotions) and difficulties with interpersonal communication [8,21,22]. These all have the potential to cause negative effects not only across the lifespan of pwHD but also their children, relatives, and friends [23,24]. In addition, features of the disease such as balance and gait issues, coupled with dysarthria, can lead to social discrimination with misperceptions from the public (e.g., that pwHD are drunk). With regard to this, it is important for us to acknowledge that the negative, external, societal responses to impairments and the active marginalisation and discrimination faced by those with visible differences or impairments (as opposed to ‘hidden’ disabilities) is much more recognised in other conditions as a source of psychological distress [25].

## 2. Clinical and Diagnostic Frameworks in HD

Despite the long-standing and well-established recognition of the wide breadth of psychological difficulties linked with HD, the most common clinical framework currently adopted to understand them appears to be biomedical—i.e., focused on identifying the cause of psychological issues in the brain changes underlying the disease [26]. For instance, this is currently reflected in the absence of psychological care within the European Huntington’s Disease Network Standard of Care Working Group guidance [27]. However, an increasing body of literature involving several other neurodegenerative conditions (e.g., Parkinson’s, amyotrophic lateral sclerosis, multiple sclerosis) has consistently shown that while biological mechanisms may certainly play a role, not all psychological distress can be explained by disease processes. Indeed, psychologically informed frameworks can be especially helpful in providing explanations based on individuals’ adjustment to being diagnosed with a life-limiting condition [25,28,29,30] as well as the rise of existential issues such as thoughts of physical and mental decline, death, and euthanasia [31]. This latter point has also been found to apply to HD, with evidence showing that low mood and cognitive impairments are more significant determinants of quality of life than motor symptoms or pain [32,33,34] and that one of the top care priorities for pwHD and their families is to receive support for the mental health aspects of the disease [35]. A number of qualitative investigations have also highlighted how psychological difficulties often thought to be caused by brain changes in pwHD (e.g., avoidance, anger) may in fact be seen as coping strategies or forms of adjustment (though admittedly sometimes maladaptively) to the disease [22,36,37,38].

Nevertheless, biomedical explanations for psychological difficulties in pwHD are still predominant, and they appear to permeate through several aspects of how we make sense of the disease and work with affected individuals. For instance, the paradigm in which research in HD is carried out is likely to affect—and may indeed explain—the number of qualitative studies exploring subjective perspectives of pwHD, particularly in comparison to other conditions [38]. However, perhaps even more importantly, the biomedical view also appears to impact people’s perceptions around their own condition, with evidence showing that pwHD tend to develop a more disease-focused view of their psychological difficulties, which is possibly due to the way these are explained to them by clinical professionals working in this biomedical framework [39].

Another crucial aspect of HD heavily influenced by biomedical perspectives is the diagnostic criteria for the condition. In particular, a positive result on the predictive genetic test has no clinical value per se, and the full diagnosis of Huntington’s disease is currently based on the presence of motor impairments as condicio sine qua non, with no requirement for cognitive impairments to be present [4]. This constitutes a shortcoming for both research and clinical practice, as in premanifest individuals, early deterioration of executive processes, working memory, and affective functioning—and consequent psychological difficulties due to adjustment—may precede the onset of motor symptoms by decades [40,41,42,43,44,45]. While the initiative to review and improve the diagnostic criteria for HD has recently started to gain momentum (e.g., [46]), this appears still far from being fully translated into everyday clinical work.

## 3. Psychological Interventions for pwHD

The current evidence around the adoption of targeted psychological interventions for pwHD and their effectiveness for different psychological outcomes is extremely sparse. In particular, to date, only one review has been carried out on the topic [47], which identified less than 10 eligible studies, only one of which was a randomised controlled trial (RCT). The studies showed some preliminary positive evidence for the effectiveness of individual cognitive–behavioural therapy (CBT), sensory modulation, progressive relaxation training, and patient education in addressing a number of psychological difficulties including anxiety, depression, and aggressiveness in manifest individuals. However, as also highlighted by recent national clinical guidance published by the British Psychological Society [48], the significant paucity of studies and high-level evidence on this topic in HD means that no formal recommendations can be made at this stage.

As a consequence, further research is strongly warranted, especially since qualitative investigations have shown that pwHD not only often express an interest in engaging with psychological interventions [39], but they also find them acceptable and beneficial when these are offered and administered [49,50]. Expert-based international clinical guidelines for treating HD also suggest that non-pharmacological interventions such as psychological therapy should be prioritised for psychological difficulties in pwHD whenever possible [51,52]. Moreover, psychological interventions are likely to play a pivotal role in addressing the several challenges linked to genetic testing, such as deciding whether (or when) to test, receiving a positive result, or testing in prenatal or paediatric settings [15].

## 4. Psychological Provision for pwHD: The Case of the UK

Even if we achieve a deeper understanding of the effectiveness of psychological interventions for pwHD, a further challenge is the successful provision of psychological care for this population. While access routes to psychological services are likely to vary greatly across different countries, no international comparisons on the availability of psychological support appear to be available to date in the literature. Therefore, the case of the United Kingdom will be presented here as an example.

Currently, the psychological provision for pwHD across the UK is patchy and unequal, with few targeted services and very few clinical psychologists with specific expertise in HD [53]. While psychiatrists can be involved in pwHD’s care, they rarely offer one-to-one therapy, instead taking on a more generic case-management role. Counselling psychologists are often involved during the predictive test period, but this support rarely continues for much longer afterwards. Individuals affected by HD who are struggling with psychological difficulties are more likely to be seen within a biomedical framework rather than a psychological one, and they are consequently often prescribed pharmacological treatments as a first-line treatment [26]. This is despite expert HD guidance recommending that psychological approaches should be considered before commencing medication for such difficulties as anxiety and agitation [50]. Although the HDA has recommended a care pathway for pwHD in the UK which includes psychology [54], the lack of established and/or ad hoc psychological care pathways also means that referrals are primarily made to psychiatry (if the consultant is a neurologist) rather than psychology, which in turn often leads to the adoption of a biomedical framework for all kinds of difficulties. This appears to be in direct contrast with a recent National Care Framework for HD produced by the Scottish Huntington’s Association, which has highlighted how clinical psychology and neuropsychology services play a pivotal role in the care of pwHD [55].

Moreover, very few individuals with HD are referred to generic mental health services in the UK. For instance, even though the Improving Access to Psychological Therapies (IAPT) programme in England has recently been widened to include long-term conditions, neurodegenerative diseases such as HD have not been actively included, leading pwHD to be often considered unsuitable for psychological services aimed at the general population [56]. PwHD are often told they need to see ‘specialist’ therapists but, as access to these is limited, they very often then feel marginalised and unsupported. While therapists with a knowledge of HD are likely to be preferred by pwHD, the active exclusion of pwHD from mainstream mental health services means that they do not receive the recommended type of therapy for those with less severe presentations of depression and anxiety [57]. Unsurprisingly, this can in turn cause a number of negative consequences for pwHD requiring specialist mental healthcare [56], and it ultimately constitutes a further obstacle to the development of an integrated and comprehensive model of care for the psychological, physical, and social needs of affected people [58].

## 5. A Five-Point Psychological Care Manifesto for People with HD

As discussed above, psychological difficulties associated with HD often have an overwhelming impact not only on affected individuals but also their immediate family and wider relationships. However, these appear to be further aggravated by the predominant biomedical framework under which they are conceptualised, a striking lack of studies exploring targeted psychological interventions, and a lack of specialised mental health care. Thus, we propose a five-point manifesto aimed at driving future improvements of the quality, effectiveness, availability, and accessibility of psychological care for pwHD. Each point is detailed below, while Table 1 provides a brief summary of their main goals.

### 5.1. Listening to People with HD

The value of qualitative methods in highlighting the subjective experiences of chronic illness, which often go overlooked in quantitative studies, is now well-established in healthcare research [59,60,61]. Issues such as ‘generalisability’, ‘validity’, and ‘replicability’, which are all perfectly valid concerns within the positivistic tradition of research associated with medicine, do not have the same relevance in qualitative research. Unless HD researchers across the research spectrum understand that quantitative and qualitative research cannot be judged by the same yardsticks, then qualitative research is always going to be seen as the less valued sibling. Journal editors need to take notice of this when selecting reviewers so that these are appropriate and knowledgeable about the particular research paradigm used. Perhaps as a reflection of the predominant paradigm in HD research, few studies have so far explored the lived experience of pwHD in general compared to other neurodegenerative conditions, and even fewer qualitative investigations are available on psychological difficulties in this population [38]. This represents a considerable limitation in our knowledge of the real-life impact of psychological difficulties in pwHD, as such investigations not only promote a more in-depth understanding of the experience of illness [38] but ultimately ensure that “patients are treated as persons and not as objects” [62] (p. 2).

As a consequence, a renewed interest in listening to the voice of pwHD and their families should represent an important priority for clinicians and researchers alike. On one hand, by recognising and embracing the value of their lived experience—and thus their role as experts in their own condition—this is meant to facilitate the expression of their views in depth in research studies and allow them to be actively involved with the planning and delivery of future research agendas. However, this also shows the potential to inform new models of care in everyday clinical practice, for instance by allowing for the challenging of unhelpful descriptions of psychological distress.

From an organisational perspective, the development of international patient engagement initiatives is likely to play a crucial role in facilitating the incorporation of the voice of pwHD in current and future clinical and research endeavours. A prime example of this is represented by HD-Cope (https://hdsa.org/hd-research/hd-cope/, accessed on 25 November 2021), a global coalition for HD patient engagement originated from a collaboration between North-American and European organisations.

### 5.2. Reformulating Difficulties Psychologically

A direct consequence of exploring the lived experience of pwHD is the opportunity to review and reformulate the current dominant explanations for psychological difficulties in HD. Far from representing merely linguistic, superficial contrasts between different clinical frameworks (e.g., biomedical vs. psychological), this process is meant to allow for the exploration of potential functional motives behind behaviours that are often attributed to underlying brain change processes. In this regard, a number of psychological constructs in HD have already been reviewed under the lens of more psychologically informed models [22,26,36,37]. For example, these have shown how avoidance may represent a strategy for pwHD to save energy in the face of chronic fatigue [22] or how irritability may represent an expression of frustration associated with other psychological difficulties such as depression and anxiety [26].

Thus, the adoption of models and frameworks from clinical, social, and health psychology (e.g., the self-regulation model [63]) should be promoted in the future to allow for the reformulation of psychological constructs in HD and to achieve a fuller understanding of psychosocial difficulties in affected individuals.

### 5.3. Exploring New Interventions

Although extremely limited, the preliminary evidence available on the adoption of CBT, sensory modulation, progressive relaxation training, and patient education appears promising and should be the subject of further empirical investigations. Similarly, further research is required to confirm the feasibility and potential effectiveness of mindfulness-based cognitive therapy (MBCT), remotivation therapy, and narrative therapy for issues such as depression, anxiety, distress, and apathy—particularly by exploring different forms of delivery such genetic counselling groups as well as online sessions [50,64,65].

Given the current paucity of literature, most psychotherapeutic approaches remain untested in pwHD at this time. However, based on positive evidence from other neurological conditions [66,67], third-wave cognitive behavioural therapies such as acceptance and commitment therapy (ACT), mindfulness-based stress reduction (MBSR), and compassion-focused therapy (CFT) may prove effective in reducing psychological difficulties among pwHD, particularly due to their focus on relaxation, the acceptance of physical impairments, and the appreciation of remaining abilities and resources [68]. In addition, given the genetic nature of HD, understanding the systemic factors at play is key [69], and therefore, family therapy or other forms of systemic therapy would be an important avenue to explore. This should also be complemented by exploratory investigations of other types of therapeutic approaches for which there is a complete lack of evidence in pwHD, such as psychodynamic psychotherapy, interpersonal therapy, or narrative therapy.

While HD represents a rare condition [70,71]—thus making large-scale studies more difficult compared to other neurodegenerative diseases—all efforts should also be made to explore the psychotherapeutic approaches outlined above with rigorous study designs yielding both quantitative and qualitative data.

### 5.4. Increasing Psychological Provision

The current situation in terms of availability of and access to specialised psychological services for pwHD across different countries remains unclear. A first step towards increasing psychological provision for this population could rely on national and cross-country surveying of mental health services in order to identify and highlight strengths and weaknesses. This applies to the UK as well, where despite the aforementioned issues, no formal survey of psychological provision for HD is currently available [48]. Qualitative investigations with individuals with HD, clinicians, and their families and caregivers can also provide invaluable insight into the subjective experience of receiving mental health care as well as potential barriers or enablers to accessing psychological services, some of which have been identified in previous patient surveys [56]. When no specialist psychological provision for HD is available or generic services could well help, efforts should also be made to allow pwHD to access mental health services aimed at the general population. This might clearly require some lobbying at a national level and the active involvement of patient support groups.

A second fundamental component of improving psychological provision is the need to increase specialist knowledge and education on HD for clinicians. This has been identified by HD families as playing a pivotal role in the successful delivery of supportive care for affected individuals [72], and it likely represents one of the cornerstones of the development of any HD specialist service. In this regard, mental health clinicians interested in HD should work alongside local and international organisations such as the Huntington’s Disease Association (HDA), the Italian League for Research on Huntington Disease (LIRH), the European Huntington’s Disease Network (EHDN), and the Huntington Study Group (HSG), which can provide specialist support, education, and training. In addition, training should be provided to interested HD clinicians in more psychological ways of thinking not only to enhance care staff’s knowledge and skills but also to allow for the development and refinement of alternative approaches to distress. Embedding psychological skills in HD multidisciplinary teams could also encourage staff to reflect on the emotional impact that working with HD can have on them and ultimately improve self-care for clinicians and caregivers.

### 5.5. Learning from Other Conditions

Considering the rarity of HD, it is perhaps not surprising that psychological care for this condition tends to be less developed compared to other neurodegenerative diseases. This is not only true in terms of research exploring the adoption of psychological interventions (e.g., see [66,67,73,74]) but also in terms of availability of specialised education and training, and accessibility of psychological provision [48,68]. While representing a disadvantage for pwHD, this disparity also shows the potential for a number of valuable lessons to be learnt from other conditions.

In particular, the current literature involving more prevalent motor neurodegenerative conditions such as Parkinson’s and multiple sclerosis—which share many of the same cognitive and psychological difficulties associated with HD [75]—offers the opportunity to draw inspiration for the adoption, refinement, and specific adaptation of existing psychotherapeutic approaches, including individual and group CBT [76,77,78], Skype-delivered MBCT and MBSR [79,80], and group-based psychosocial therapy [81]. Similarly, the key competences and expertise required for the development of specialised psychological provision may be obtained by liaising with local and international organisations dedicated to other conditions, such as the Michael J Fox Foundation, the European Parkinson’s Disease Association (EPDA), and the Multiple Sclerosis International Federation.

## 6. Conclusions

Huntington’s disease is a rare and complex condition that imposes a number of long-term and fluctuating challenges on affected individuals, family members, caregivers, and clinicians alike. Due to the predominant biomedical framework adopted with HD, which has traditionally viewed it as a brain disorder first and foremost, one of the most overwhelming of these challenges—psychological care—is often overlooked in everyday clinical practice.

In this paper, we have proposed a manifesto delineating five points to address the quality, effectiveness, availability, and accessibility of psychological care in this population. These include (1) Listening to People with HD, (2) Reformulating Difficulties Psychologically, (3) Exploring New Interventions, (4) Increasing Psychological Provision, and (5) Learning from Other Conditions.

As the search for a cure continues, we hope that this manifesto will drive a new impetus towards refining the current approach to psychological difficulties in HD, seeing it as more than just a brain disorder and ultimately improving the quality of life of the tens of thousands of families affected by HD worldwide.

## Figures and Tables

**Table 1:** Summary of the manifesto’s points and goals.

Point	Goals
Listening to People with HD	Carrying out more qualitative studies exploring the subjective experience of pwHD. Allowing pwHD and family members to express their views fully and in depth in clinical and research settings. Facilitating the challenging of unhelpful descriptions of distress as inevitable and without hope for help. Recognising pwHD’s role as experts in their own condition. Promoting the development of international patient engagement initiatives to facilitate patient/carer involvement in the research agenda setting.
Reformulating Difficulties Psychologically	Reviewing psychological constructs in HD under the lens of more psychologically informed models. Integrating models and frameworks from clinical, social, and health psychology. Focusing on the functional nature of behaviours and the potential motives behind them.
Exploring New Interventions	Carrying out research on the effectiveness of CBT, sensory modulation, progressive relaxation training, and patient/carer education. Confirming the feasibility and potential effectiveness of MBCT, remotivation therapy, and narrative therapy. Exploring other third-wave therapy approaches such as ACT, MBSR, and CFT. Exploring family therapy or other forms of systemic therapy in response to the hereditary nature of HD. Exploring therapeutic approaches which are currently neglected in pwHD (e.g., psychodynamic, interpersonal, and narrative therapy).
Increasing Psychological Provision	Surveying national psychological provisions for HD and performing cross-country comparisons. Carrying out qualitative studies with pwHD, clinicians, family members, and caregivers to identify barriers and enablers to accessing psychological services. Allowing pwHD to access generic mental health services when no specialist psychological provision is available. Increasing knowledge and education on HD for clinicians by liaising with HD organisations. Enhancing HD care staff’s psychological knowledge and skills to develop and refine alternative approaches to distress and promote staff self-care.
Learning from Other Conditions	Learning lessons from more prevalent neurodegenerative diseases that share similar difficulties with HD. Drawing inspiration from psychological interventions adapted for Parkinson’s and multiple sclerosis (e.g., CBT, MBCT, MBSR, psychosocial therapy) to drive HD-focused adaptations. Liaising with other diseases’ organisations to draw key competences and know-how required for the development of specialised psychological provision.

Note. ACT = acceptance and commitment therapy; CBT = cognitive–behavioural therapy; CFT = compassion-focused therapy; HD = Huntington’s disease; MBSR = mindfulness-based stress reduction; MBCT = mindfulness-based cognitive therapy; pwHD = people with HD.
